# Critical national response in coping with Omicron variant in China, Israel, South Africa, and the United States

**DOI:** 10.3389/fpubh.2023.1157824

**Published:** 2023-06-09

**Authors:** Jun Jiao, Leiyu Shi, Haiqian Chen, Xiaohan Wang, Manfei Yang, Junyan Yang, Meiheng Liu, Gang Sun

**Affiliations:** ^1^Department of Health Management, School of Health Management, Southern Medical University, Guangzhou, Guangdong, China; ^2^Department of Health Policy and Management, Bloomberg School of Public Health, Johns Hopkins University, Baltimore, MD, United States

**Keywords:** COVID-19, Omicron variant, containment strategy, mitigation strategy, national response

## Abstract

**Objective:**

The aim of this study was to analyze the effectiveness of prevention and control strategies and put forward further measures according to the epidemiological characteristics of Omicron. It summarized the national response during the Omicron epidemic in four countries: China, Israel, South Africa, and the United States.

**Methods:**

This study summarized prevention and control measures in China, Israel, South Africa, and the United States in their response to the Omicron epidemic, and it also evaluated the effectiveness of these measures.

**Results:**

After the Omicron variant emerged, China and Israel adopted containment strategies, using the “dynamic zero” policy and country closure measures. Meanwhile, South Africa and the United States adopted mitigation strategies, which virtually abandoned social interventions and only focused on medical measures and vaccines. From the first day of reported Omicron cases to 28 February 2022, the four countries reported the following cases: China reported 9,670 new confirmed cases and no deaths, with total deaths per million of 3.21; Israel reported 2,293,415 new confirmed cases and 2,016 deaths, with total deaths per million of 1,097.21; South Africa reported 731,384 new confirmed cases and 9,509 deaths, with total deaths per million reaching 1,655.708; the United States reported 3,042,743 new confirmed cases and 1,688,851 deaths, with total deaths per million reaching 2,855.052, which was much higher than the other countries.

**Conclusion:**

Based on this study, it seems that China and Israel adopted containment strategies, while South Africa and the United States adopted mitigation strategies. A rapid response is a powerful weapon against the Omicron epidemic. Vaccines alone will not get any country out of this crisis, and non-pharmacological measures should be used in addition to them. According to the SPO model, future work should consider the strengthening of emergency management capacity, adhering to public health measures, promoting vaccination, and strengthening patient care and close contact management, which are effective measures in coping with Omicron.

## Introduction

COVID-19 continues to pose a very high health threat worldwide, and waves of the COVID-19 epidemic are occurring one after another due to mutated variants. After the Alpha, Beta, Gamma, and Delta SARS-CoV-2 variants of concern (VOC), on 24 November 24 2021, South Africa reported to the World Health Organization (WHO) the discovery of a new SARS-CoV-2 variant B.1.1.529, which was named Omicron after 2 days (1). The Omicron variant had an overall global risk assessment of “very high” and spread worldwide (2). By 28 February 2022, there were 4,34,154,739 confirmed cases worldwide and 5,944,342 deaths.

The Omicron variant has important amino acid mutation sites for all previous four VOC variants that spur proteins, including mutation sites that enhance cellular receptor affinity and viral replication ability (3). Omicron spreads faster, and it is roughly 10 times more infectious than the original virus and twice as infectious as the Delta variant (4). Omicron has increased transmissibility, with the ability to evade immunity conferred by past infection or vaccination. Thus, Omicron generally causes less severe disease than infection with prior variants, which could threaten the effectiveness of current vaccination measures (e.g., reducing the effectiveness of current diagnostics, vaccines, and therapies) (3, 5). In addition, immunity duration after infection is about 3–6 months, and the variability of Omicron leads to a low rate of severe illness but increases the risk of re-infection.

From November 2021, the BA.2 subtype began to spread in India, South Africa, and the Philippines. The transmission rate of BA.2 is about 30% higher than BA.1, and more than 90% of infections are asymptomatic infections. There are 20% of latent infections (patients are undetectable by nucleic acid testing), which poses new challenges for detection and traceability (6, 7). In Jan 2022, the proportion of BA.2 sequences even exceeded that of BA.1 in Botswana, Denmark, Sweden, and India.

The spread of Omicron has been met with different responses from different countries. China is an importer of Omicron. Since January 2022, there have been indigenous epidemics caused by Omicron in Tianjin, Anyang, and Baise. The Omicron epidemic was responsible for the largest wave of infections in China after the first wave of COVID-19. Israel, a “vaccination honoree,” entered a violent fifth wave of the epidemic in December 2021, becoming the first closure country under Omicron. South Africa, the first country to report the Omicron variant, adopted a combination of mitigation and containment strategies in response to the previous three waves of COVID-19, but it began to resume work and production prematurely. The United States was severely affected by Omicron, where its rapidly increased, and on 9 January 2022, the Omicron variant accounted for about 98% of total cases, and hospitalizations underwent a 24.5% increase from the previous 7 days (8).

Vaccines are considered the key measure in combating COVID-19, and several studies show that vaccines can prevent severe illness and death. However, in some African countries, vaccination remains a privilege for a minority of the population (9). Omicron led to a dramatic increase in cases not only in countries with low vaccination rates, such as South Africa and Botswana, but also in countries with high vaccination rates, such as Israel and the United Kingdom, where a new COVID-19 wave began. Due to the variability and immune escape of Omicron, vaccination rates have not reached the herd immunity threshold, and with the shortage of vaccines in low-income countries and the time frame for vaccine protection, vaccines alone will not get any country out of this crisis (10). Countries should combine vaccination and non-pharmaceutical interventions, analyze the form of epidemic risk in time, and develop appropriate national response strategies (11, 12). To analyze the effectiveness of prevention and control strategies and put forward further measures according to the epidemiological characteristics of the Omicron variant, this study summarized the national response during Omicron epidemic in four countries: China, Israel, South Africa, and the United States.

## Methods

### Measures collection

We searched for COVID-19 prevention and control measures from office websites between the day of the first reported Omicron cases and 28 February 2022. The selection criteria and search strategy were as follows: (1) the measures prepared for COVID-19; (2) the measures changed after the discovery of the Omicron variant; (3) the measures were non-pharmaceutical interventions; (4) the measures were issued by the national government or state government.

A systematic literature search was performed in the National Health Commission of the People’s Republic of China,^1^ Israel’s.

Ministry of Health,^2^ South Africa’s National Department of Health,^3^ and the Centers for Disease Control and Prevention of the United States,^4^ in which literature was collected and screened following defined criteria for analysis. The last search was run on 1 April 2022.

### Data collection

The COVID-19 data were extracted from official websites and updated in real time, including the Coronavirus Resource Centre at Johns Hopkins University^5^ and WHO Coronavirus (COVID-19) Dashboard.^6^ This study investigated new confirmed cases, total cases, total deaths, and total cases per million, which take country-specific conditions and population bases into account in China, Israel, South Africa, and the United States from the day of the first reported Omicron cases to 28 February 2022.

All in all, this study selected COVID-19 data and the major measures carried out between the day of the first reported Omicron cases and 28 February 2022 and analyzed the effectiveness of the COVID-19 major measures during the Omicron epidemic. It also assessed the effectiveness of controlling COVID-19 cases in four countries by combining total new cases and total new deaths during the Omicron epidemic.

## Results

### National strategy in response to omicron variant

#### Containment strategy

Both China and Israel are importers of the Omicron variant, and both countries quickly took border prevention and control measures after the first Omicron case. China has continued its ongoing entry control measures, implemented closed-loop management of arrivals, and issued risk alerts at border crossings. Israel closed its international airport and began a country-wide closure on 26 November 2021 after reporting the first Omicron case, becoming the first country to close the country due to the Omicron variant.

China is not only facing Omicron but also facing the threat of Delta. China adheres to the “guarding against imported cases and preventing a resurgence of epidemics in domestic” strategy and “dynamic zero” policy. Rather than adopting a uniform national strategy for epidemic prevention and control, China has adopted a “targeted management” strategy in different areas. On 8 January 2022, two Omicron cases were reported in Tianjin. As for movement restrictions, Tianjin issued control measures of “not leaving Tianjin unless necessary” and cut off passenger services in some areas; as for social distancing, Tianjin closed educational institutions, adopted closed management, and restricted gathering; and as for public health measures, Tianjin adopted multiple rounds of full staff nucleic acid testing and enhanced virus traceability and early warning of detection at key sites. In the treatment of the Omicron variant, Tianjin’s emphasis has been placed on both Chinese and Western medicine, with Chinese medicine participating in the medical treatment of confirmed cases throughout the whole process. The Omicron transmission period coincided with an important traditional festival, the Chinese New Year. China prepared emergency plans in advance and strengthened the communication and collaborative response mechanism of various departments.

On 26 November 2021, the Israeli Ministry of Health issued a statement saying that the country’s first Omicron case had been identified. The statement said the case came from the African country of Malawi. In addition, two other people entering the country were are also suspected of having been infected with Omicron. Israel quickly closed its borders, switched schools to online teaching, and approved several prevention and treatment drugs. In addition, it used the traffic light model to show the extent of the regional epidemic. According to characteristics of the Omicron variant, it shortened the isolation period and replaced nucleic acid testing with antigen testing. Israel was the first country to introduce a third-dose vaccination and to focus its vaccination efforts on the protection of vulnerable populations. However, on 1 March 2022, Israel lifted almost all social interventions and fully opened its borders to all negative travelers, regardless of their vaccination status. Table 1 summarizes the major measures taken by China and Israel in response to the Omicron variant, including movement restrictions, governance and socioeconomic measures, social distancing, and public health measures.

**Table 1:** The major measures taken by China and Israel in response to the Omicron variant.

Countries measures	China	Israel
Movement restrictions	On 20 December 2021, border cities were required to limit tourism, alert risks, and reduce tourist population to avoid cluster epidemics. On the evening of 8 January 2022, the Tianjin Epidemic Prevention and Control Headquarters implemented strict control measures stating that no one would leave Tianjin unless necessary. On 10 January residents in Anyang, Henan province, were ordered to keep vehicles off the roads and stay indoors. Implementation of the whole closed-loop management of inbound personnel; personnel control in high-risk positions; increase of sample inspection of imported goods; implementation of disinfection measures for entry aircraft. When there are cluster epidemics, cities should cut off external passenger services and strengthen urban passenger transport management. Port staff adopt the contactless declaration.	On 29 November 2021, Israel’s only international airport, Ben Gurion International Airport, was closed for two weeks, barring foreigners from entering. Israel designated several countries as “red” high-risk countries, barring its citizens from visiting. On 9 December, Israel announced that it was extending travel restrictions by 10 days, barring all foreigners from entering the country until 22 December. During this period, Israelis entering the country had to undergo nucleic acid testing and isolation. Israelis were subject to quarantine for a period between 3 and 14 days upon entry, depending on whether they had completed vaccination or recovered from COVID-19 infection. The government announced that it would fully open the border to travelers who had not been vaccinated on 1 March 2022.
Governance and socioeconomic measures	The NHC strengthened the monitoring and information reporting of COVID-19 and the implementation of “every positive must be reported in time, receiving of the report must be the check-in time.” The workgroup carried out emergency response work in provinces at risk of epidemic spreading. On 7 January 2022, the NHC issued a work plan, which established a collaborative mechanism, shared information in a timely manner, and carried out daily scheduling to strengthen emergency preparedness for epidemic prevention and control during the Spring Festival.	On 16 January 2022, the Israeli cabinet approved an increase in state aid to airlines affected by COVID-19 to help them weather the storm. The government approved the extension of the state of emergency until 20 February 2022.
Social distancing	On 9 January 2022, training institutions, offline training, and hosting services were suspended. In Tianjin, interviews for teacher qualification examinations were canceled, some subway stations were temporarily closed, and many scenic spots were suspended. From 12 January, primary and secondary schools in Tianjin had their winter vacation extended, and all schools implemented closure management. Reducing gatherings, strictly controlling large-scale events according to the principle of “not holding events unless necessary,” and maintaining social distancing and other protective measures.	On 21 December 2021, Israel introduced measures such as restricting the flow of people in shopping malls and other large public places and switching schools in hard-hit areas to distance learning. On 20 January 2022, children under the age of 18 would not need to be quarantined if they tested three times within 5 days of coming into contact with a confirmed patient. Since 27 January, all students received nucleic acid tests twice a week, and if they were positive, they were required to stay home until they recovered.
Public health measures	Preventing the outflow of epidemics: the infected area carried out virus traceability investigation, centralized isolation, home management of control areas, restricted movement, and suspended gathering activities to cut off the transmission chain. On 9 January 2022, Tianjin launched full nucleic acid testing for all residents of four districts within 24 h, and from 10 January, all residents of the other 12 districts were tested within 24 h. On 12 January, a half-day holiday took place for the second round of nucleic acid testing. All regions continued carrying out surveillance and early warning of key populations, key sites, and key units, guiding the public to adhere to daily health protection, and quickly handling each cluster epidemic. All regions strengthened cold chains and other logistics management, required strict implementation of disinfection and ventilation, established employee protection, information registration, and a staff health abnormal reporting system.	Nucleic acid testing requirements were adjusted so that groups aged 60 years and older and those with poor immune function would take nucleic acid tests, while groups at low risk of infection only received rapid antigen tests. Vaccinated or recovered people from a previous infection could take a rapid antigen test at home after contact with a confirmed case. If the test was negative, they could live at home normally. If the test was positive, they would be quarantined for 10 days. If unvaccinated people were to come into contact with a confirmed case, they were required to undergo a test and quarantine for 7 days or 10 days. On 30 December 2021, masks were required be worn by groups of more than 50 people. On 11 January 2022, the MOH reduced the isolation period for confirmed cases from 10 days to 7 days, and on 17 January, it was reduced to 5 days. According to the traffic light model, Israel was divided into red, orange, yellow, and green areas. Evaluation policy, prevention policy, and fatal treatments were to return to normal. The Israeli police was preparing for a significant tightening of the enforcement of the Green Pass policy.
Medical measures	Specific nucleic acid detection methods were established for Omicron, and viral genomic surveillance of imported cases has been ongoing. China has advanced vaccines (booster dose for 3–17 years old and adults) and deployed sequential booster immunization. The specification for a nucleic acid 20-in-1 mixed detection technique was published, and the price of nucleic acid testing was lowered. Nucleic acid testing sites were opened for convenience. China insists on medical treatment gate-shifting and the combination of Chinese and Western medicine, and patients are diagnosed and examined by expert groups, while individualized treatment plans are formulated, and homogeneous treatment is carried out.	In December 2021, the MOH approved Paxlovid, an anti-viral drug by Pfizer for the treatment of COVID-19. In January 2022, the MSD approved Merck’s molnupiravir, an anti-viral for the treatment of COVID-19. In February, AstraZeneca approved its coronavirus prevention drug Evusheld. Israel launched its fourth dose of COVID-19 vaccine for people over 60 years old and health workers on 6 January 2022. Israel’s MOH approved the fourth dose of the COVID-19 vaccine for the country’s immunocompromised population on 30 December 2021.

NHC, National Health Commission of the People’s Republic of China; MOH, Israel Ministry of Health.

#### Mitigation strategy

The Omicron variant was first detected in South Africa on 9 November 2021. By 1 December, for a month-long period, South Africa had little policy response to the Omicron. By mid-December, it began closing schools, adjusting the national statement, and closing nightclubs and some venues. On 30 December, the government said that the COVID-19 data showed that the country had passed the fourth wave peak of the epidemic and would lift curfews and limit gathering. Subsequently, South Africa stepped up vaccine procurement, closed some borders, and resumed classes. It also announced that isolation would be recommended for asymptomatic infections rather than mandatory isolation and allowed participation in social activities, which only required the wearing of masks. From 23 February 2022, South Africa shortened vaccination schedules and allowed different brands of vaccines to promote vaccination.

The United States, which is an importer of the Omicron variant, implemented entry restrictions for southern African countries on 26 November 2021 and added a negative nucleic acid test certificate for international travelers on 6 December; subsequently, international travelers were advised to go into self-isolation upon entry. On 1 December, the United States reported that California had identified its first Omicron case, the carrier of whom had returned to California from South Africa on 22 November and tested positive on 29 November. The United States declared an extended national emergency, introduced indicators for monitoring COVID-19 community levels, and implemented prevention strategies. In the face of the skyrocketing rate of confirmed cases and insufficient medical resources, the United States encouraged people to conduct self-testing at home and shortened the isolation period based on the Omicron incubation period, as well as promoting vaccination, strengthening public health measures such as wearing masks and increasing physical distance in schools, and adopting measures—such as permitting healthcare workers with asymptomatic COVID-19 to return to work after 7 days with a negative test—and other measures to alleviate the shortage of healthcare workers. Table 2 summarizes the major measures taken by South Africa and the United States in response to the Omicron variant, including movement restrictions, governance and socioeconomic measures, social distancing, and public health measures.

**Table 2:** The major measures taken by South Africa and the United States in response to the Omicron variant.

Countries measures	South Africa	United States
Movement restrictions	On 11 January 2022, South Africa closed all 20 land border crossings that were open, including Beit Bridge with Zimbabwe and Maseru Bridge with Lesotho. On 13 February, South Africa’s borders with Zimbabwe, Mozambique, Namibia, Lesotho, Eswatini, and Botswana were reopened.	On 26 November 2021, a presidential proclamation suspended entry into the United States for non-citizens who were present in the eight countries in southern Africa during the 14 days preceding travel to the United States. On 6 December, international travelers entering the United States were required to present a negative test within 24 h. The CDC recommended that all travelers should obtain a COVID-19 viral test 3–5 days after arrival, and travelers who were not fully vaccinated should self-quarantine for 7 days, even if their test result was negative.
Governance and socioeconomic measures	Since 30 Dec 2021, South Africa has been on adjusted alert level 1. The government is continuing to provide social relief distress grants. The Special COVID-19 Social Relief of Distress amount is R350 per month from the date of approval. A person may not be evicted from their land or home or have their place of residence demolished for the duration of the national state of disaster. The government has run a coordinated public information campaign.	Local health authorities and community coalitions should identify additional temporary housing and shelter sites that can provide appropriate services, supplies, and staffing. The Biden administration required private health insurers to fully reimburse their customers for over-the-counter nucleic acid tests. President Biden said that the national emergency declared in March 2020 due to COVID-19 would be extended beyond 1 March due to the ongoing risk to public health.
Social distancing	Between 16 December 2021 and 12 January 2022, schools were closed for the academic break. On 7 February, schools resumed full-time classes. On 30 December 2021, places and premises were closed to the public. Night vigils were not allowed. Businesses were required to follow health protocols and social distancing measures, as well as sector-specific restrictions. Nightclubs were closed to the public. On 30 December, gatherings were restricted to no more than 1,000 people indoors and no more than 2,000 people outdoors.	In addition to universal indoor masking, the CDC recommended that schools maintain at least 3 feet of physical distance between students within classrooms. Shared housing dormitories in institutions of higher education were considered a lower risk congregate setting due to the lower risk of severe health outcomes associated with young adults. Therefore, the CDC recommended that shared housing in IHE settings follow the general public guidance for quarantine and isolation.
Public health measures	On 30 December 2021, measures such as curfews and restrictions on attending outdoor and indoor activities were lifted. On 1 February 2022, asymptomatic infected persons would not have to be quarantined but were advised to do so. They were required to wear masks for 5 days, avoid social gatherings, and, in particular, avoid socializing with the older adult and those with complications. Those with mild illness were required to quarantine for 7 days. Nucleic acid testing was not required until the lifting of the quarantine. Tracing of all quarantined and close contacts ceased. On 1 February 2022, the curfew was be lifted, with no restrictions on the hours of movement of people. Masks were required to be worn in public places Comprehensive contact tracing of cases continued to take place.	A COVID-19 community levels tool was developed to help communities decide what prevention steps to take based on the latest data. Airport surveillance of post-arrival testing and sequencing: there was voluntary testing for international passengers, and people were encouraged to conduct nucleic acid self-tests at home. Everyone aged 2 years or older required to wear masks in public indoor places in areas of high transmission, and unvaccinated people required to wear masks. The COVID-19-infected person and close contacts were required to follow existing CDC guidance on when and how long to self-isolate. On 4 January 2021, the CDC updated COVID-19 isolation recommendations with shorter isolation periods of 5 days to focus on the period when a person is most infectious. On 3 March 2022, the CDC stated that more than 90% of the United States population lived in areas where masks were no longer required, which applied to everyone, including school children and unvaccinated people.
Medical measures	On 23 February 2022, South Africa shortened the interval between vaccinations (from 42 days to 21 days) to allow “mixed vaccinations” of different brands of the vaccine. The DOH rushed to procure vaccines and announced that everyone aged 12 years and older was eligible for the vaccine. The cost was fully covered by the government. The DOH also deployed a large number of health officials to communities to set up temporary vaccination sites and implement compulsory vaccination measures.	On 4 November 2021, a mandatory vaccination order was issued, requiring all companies with more than 100 employees to fully vaccinate and weekly nucleic acid tests for employees who did not vaccinate. The CDC advised everyone aged 16 years and older to get a booster shot vaccine. An additional 50 million free tests were provided for the uninsured. Laboratory testing and surveillance for the rapid confirmation of presumptive variants’ characters were strengthened. Asymptomatic healthcare workers were permitted return to work after 7 days with a negative test, and their isolation time could be cut further in the event of staffing shortages. Healthcare workers who received all vaccine doses were not required to quarantine following high-risk exposures.

DOH, Department of Health, Republic of South Africa; CDC, The Centers for Disease Control and Prevention.

### Results of national strategies in response to omicron variant

**Figure 1:**
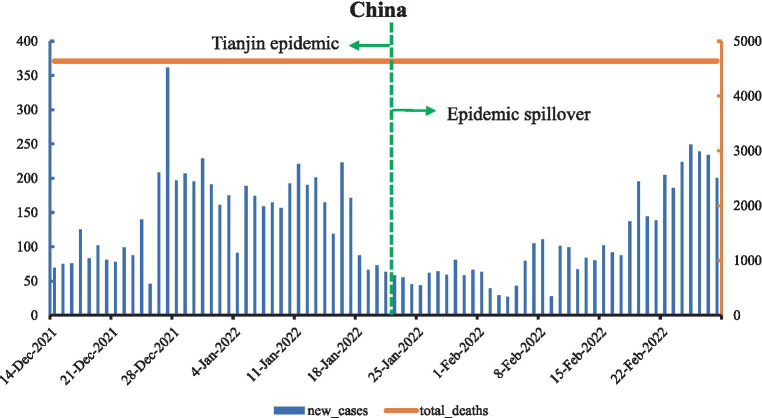
Curve of Omicron variant of COVID-19 epidemic in China.

As shown in Figure 1, on 13 December 2021, an Omicron case was reported in Tianjin, which was the first reported Omicron variant case in mainland China. The Omicron variant led to the most confirmed cases in China since the Wuhan epidemic in 2019. Initially, Omicron cases were mainly concentrated in Tianjin, but in January 2022, the Omicron epidemic spilled over to Dalian, Liaoning, and Anyang, Henan, with the second Omicron wave outbreaks. On January 21 2022, Tianjin declared the epidemic to be “socially cleared” as part of the first large-scale battle against Omicron in China. New confirmed cases decreased and remained between 50 and 100 cases. In February, Omicron cases were reported in many places, and new confirmed cases started to grow, with a total of 9,670 new confirmed cases and no deaths during this period and total deaths at 3.21 per million.

**Figure 2:**
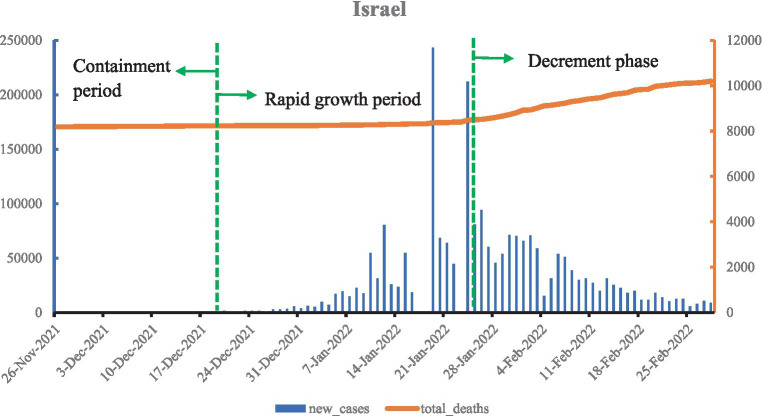
Curve of Omicron variant of COVID-19 epidemic in Israel (data vacancy exists on 17, 18, and 23 January 2022).

As shown in Figure 2, the Omicron epidemic in Israel can be divided into three phases based on new confirmed cases: the containment period, rapid growth period, and decline period. During the containment period, the Omicron variant began to spread as soon as it was reported, with new cases stabilizing to under a thousand. Starting from mid-December 2021, new confirmed cases began to grow rapidly, surpassing 1,000 cases on 20 December 2021, exceeding 10,000 cases on 5 Jan 2022, and peaking on 19 January. Thereafter, new confirmed cases gradually fell back into the decline period. Unlike China, deaths in Israel increased with the new confirmed cases, and by 28 February, there were 2,293,415 new confirmed cases and 2,016 deaths, with total deaths per million reaching 1,097.621.

**Figure 3:**
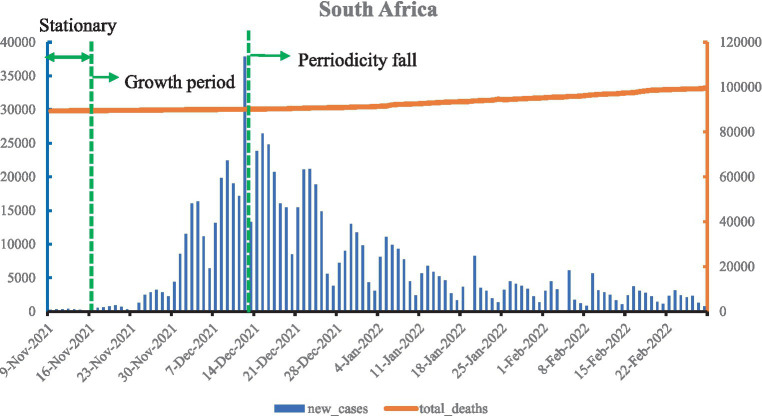
Curve of Omicron variant of COVID-19 epidemic in South Africa (data vacancy exists on 23 November 2021 and 18, 19, and 24 February 2022).

As shown in Figure 3, when the Omicron variant was detected in South Africa on 9 November 2021, new confirmed cases remained stable for only half a month. A dramatic increase began on 24 November, and the South African Department of Health announced the official fourth wave of the epidemic peak on 3 December, with the peak reaching more than 37,000 new confirmed cases on 12 December. The new confirmed cases in South Africa show a cyclical trend, with a post-peak period followed by a fall and another peak. In addition, the number of deaths shows a uniform increase. By 28 February 2022, the total confirmed cases were 731,384 and deaths increased by 9,509, with total deaths per million at 1,655.708.

**Figure 4:**
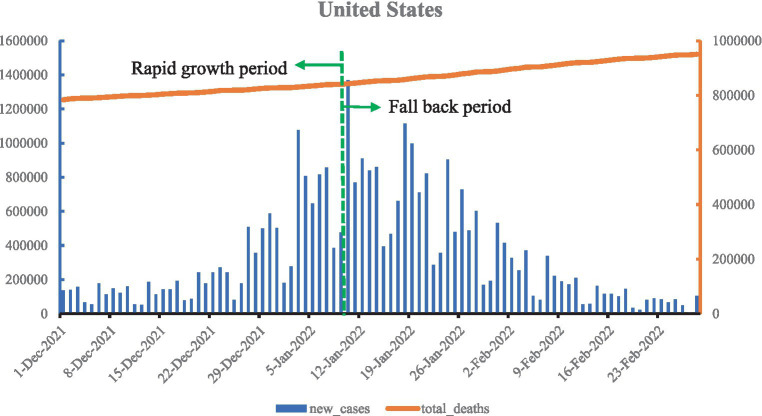
Curve of Omicron variant of COVID-19 epidemic in the United States.

As shown in Figure 4, Omicron spread in the United States, accounting for 92.3% of new confirmed cases in the week of 1 January 2022, and new confirmed cases also increased, slightly higher than previously. In January, the Omicron variant spread more widely across the country. On 10 February, new confirmed cases reached a staggering 1.36 million, a record high, marking the Omicron epidemic peak, after which new confirmed cases fell back. Deaths continued to climb, and by 28 February, the Omicron epidemic had accumulated 30,462,743 new confirmed cases and 168,851 deaths, with total deaths per million at 2,855.052.

**Figure 5:**
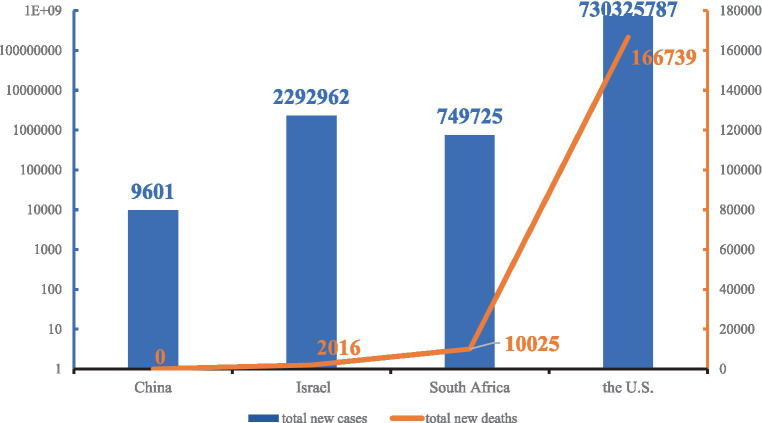
Total new cases and total new deaths in China, Israel, South Africa, and the United States during the Omicron variant of the COVID-19 epidemic.

The results in Figure 5 comprise the number of COVID-19 cases on 28 February 2022 subtracted from the COVID-19 cases on the day of the first reported Omicron cases. As shown in Figure 5, during the Omicron epidemic, the total new cases and total new deaths in China, Israel, South Africa, and the United States increased in sequence. China increased its total number of new cases by 9,601 and maintained zero new deaths. Israel’s total new cases (2,292,962) were higher than South Africa’s (749,725) but the total new deaths (2,016) remained lower than South Africa’s (10,025). The total new cases and total new deaths in the United States increased exponentially to 730,325,787 and 166,739, respectively.

## Discussion

Scholars around the world are debating whether Omicron means that the COVID-19 epidemic is coming to an end or marks the start of a new global pandemic. The WHO Director-General, Dr. Tedros said it was “wrong for people to consider Omicron as mild” (13). According to the WHO, Omicron is more transmissible and has been shown to produce breakthrough infections. In terms of virulence, the death risk caused by Omicron is 69% lower than Delta, but Omicron is 6–9 times more contagious than influenza and is a breakthrough infection, and thus it may cause more infections and deaths (14). All over the world, no health system can withstand the COVID-19 pandemic, and existing vaccines are less effective against Omicron’s neutralizing antibodies, meaning there are higher requirements for the rapid implementation of non-pharmaceutical interventions. Thus, Omicron remains serious (15).

At this point, there are two directions for coping with Omicron. One is the containment strategy, which firmly takes measures to contain the spread of the epidemic, and the other is the mitigation strategy, which abandons epidemic prevention and control measures. Omicron spreads very rapidly, and the current level of epidemic prevention is insufficient. Therefore, there is no chance to regain control once epidemic prevention measures are abandoned, and a government’s judgment and choice of prevention policies will affect the health and lives of people in their country and even the world. This study discusses two response strategies adopted by four countries and makes recommendations for further measures to combat the Omicron pandemic.

### Containment strategy

The goal of the containment strategy is to stop the virus from spreading as much as possible and eventually make the epidemic disappear. This is manifested by actively identifying cases and taking action, especially actively tracking and isolating close contacts to cut off the transmission route. Both China and Israel adopted containment strategies, and China has been practicing a dynamic zero policy of detecting one case and containing one case. Israel quickly closed its borders and country.

Omicron in China is characterized by “many points, extensive and frequent,” and the epidemic is very serious. China adhered to the “dynamic zero” policy, which means that when a case or an epidemic emerges, it can be quickly detected, quickly contained, and its transmission process cut off, thus finally achieving the “discover one, extinguish” approach in order to prevent the epidemic from causing community transmission. After discovering an outbreak, the National Health Commission of the People’s Republic of China would immediately send a task force to participate in prevention and control. However, the “zero policy” has been questioned. Some scholars argue that a blockade is not a long-term solution and that the country should be opened. China is the only country that has achieved positive GDP growth and completed poverty eradication in 2020. Confirmed cases in China were only 0.0076% of the country’s population, and the death rate was very low. If intervention measures are lifted, according to China’s population base and population density, infections may grow exponentially, which will easily cause a run on medical resources with unimaginable consequences. At this stage, it is still necessary to adhere to the “zero policy” and gradually open up, exchanging a short period of embargo for a long period of normal life (16).

Israel is known as a “vaccination honoree” and the first country to introduce a third-dose vaccine. Before the Omicron epidemic, Israel was actively preparing for the implementation of a fourth-dose vaccine. When the first Omicron case was reported, over 60% of Israel’s people were fully vaccinated (13). Due to the early vaccinations, the protective effects of the vaccines have decreased, and new confirmed cases remain at a high level (17–20). Israel opened its borders on 1 March 2022, marking the first time since the COVID-19 epidemic that it fully opened its borders. In addition, Israel has adjusted its coronavirus detection requirements of rapid antigen testing for groups at lower risk of infection, replacing the $10.08 PCR test with a $5.70 rapid antigen test, in part to reduce the cost of coronavirus testing (21). The Israeli experience shows that a high rate of vaccination may not prevent the infection and transmission of Omicron, but it can effectively reduce severe illness and mortality and keep the healthcare system from collapsing. Moreover, high vaccine coverage can help control the epidemic and restore normalcy to life.

### Mitigation strategy

The mitigation strategy is also used in response to a pandemic, and it is recommended by the WHO for use in phases 5 and 6 of a pandemic, primarily to reduce its impact and contain an epidemic within a certain range. If mitigation strategies are implemented, vaccine promotion and strengthening the carrying capacity of the health system become very important. In response to Omicron, South Africa and the United States did not strengthen their epidemic measures but rather shortened their isolation times, relaxed mandatory isolation, and focused their epidemic prevention on vaccination and severe case management.

By November 2021, after the third wave of COVID-19 in South Africa, seroprevalence was 60% in a rural community and 70% in an urban community (22). The “asymptomatic carriage” rate of Omicron is much higher than prior variants. Significant asymptomatic infections, coupled with the insidious nature and the small amount of viral excretion during the incubation period, make it difficult for detecting the disease, and a single nucleic acid test may show a false negative. During the Omicron epidemic, South Africa reached the peak of the epidemic faster than in the previous three waves, but the disease was milder, and the risk of serious illness was 30% lower than in prior variants (23). On 30 December, the South African government announced that the country had passed the peak of the fourth wave of the epidemic (24). The WHO announced on 18 January 2022 that in South Africa the Omicron epidemic was coming to an end (25). South Africa began to liberalize its borders, resume schooling, lift restrictions, and lift mandatory quarantine of asymptomatic infections, shifting from a blockade to improving population immunity. However, South Africa has a young population and a high early infection rate, which may have contributed to the mild impact of the epidemic. From the global data, the COVID-19 pandemic is still not over, and the BA.2 variant is gradually becoming the dominant virus.

Compared to the death rate in South Africa, daily new deaths in the United States remained at a peak after the previous rounds of confirmed cases and deaths. By February 2022, even with more than 900,000 total deaths, there were still about 2,000 deaths per day (26). The United States COVID-19 response has been called “living with COVID-19,” and no effective prevention policy has even been proposed so far. Rather, it opted to eliminate masks, shorten quarantine times, and slow down prevention policies (27). With the following spike in new confirmed cases, the run on the United States healthcare system was severe. To alleviate the strain on healthcare workers, asymptomatic infected healthcare workers were permitted to return to work after 7 days, and vaccinated healthcare workers do not need to be quarantined even after high-risk exposures. Studies show that United States health workers, especially nurses and advanced practice providers, are experiencing COVID-19-related psychological distress (28). In terms of the healthcare system, it is not enough to ignore epidemic prevention measures and focus solely on vaccination and treatment of serious cases. Healthcare workers and medical equipment are severely overloaded, and nucleic acid testing capacity is insufficient, resulting in confirmed cases not being effectively treated and accelerating transmission, thus creating a vicious cycle (29, 30).

### Further measures

As the world enters the third year of the COVID-19 epidemic, after four or five waves of the epidemic, many countries are gradually giving up on prevention. However, the Omicron variant has an average basic and effective reproduction number of 8.2 and 3.6, which means that it has a strong spread (31). Moreover, more and more countries’ epidemic data tell us that vaccines alone will not get any country out of this crisis (10, 32). With the current level of epidemic prevention and capacity of healthcare system, once the epidemic invention measures are abandoned and the optimal period of intervention for Omicron variant is missed, there will be no chance to regain control; thus, countries should take active and effective intervention measures to cope with the Omicron variant.

This study introduces the structure–process–outcome (SPO) model, which was first introduced by the American scholar Avedis Donabedian in the Evaluation of Health Care Quality, which proposes COVID-19 prevention and control measures to cope with the Omicron variant from three dimensions: structure, process, and outcome (33). In the prevention and control of COVID-19, the “structure” dimension includes emergency plans for public health emergencies, governmental epidemic networks, emergency command systems, regulations, and resource allocation, which are mainly reflected in governance and socioeconomic measures. The “process” dimension includes the general protection of the population, mainly reflected in movement restrictions, social distancing, and public health; the “outcome” dimension includes the treatment of confirmed cases and the management of close contacts and experience summaries, covering medical measures and other components (34–36).

#### Structure

Firstly, countries around the world have to cooperate to fight COVID-19. COVID-19 has become a global pandemic, and no country can stay out of it (37). Secondly, the Omicron variant is always mutating, from the original strain BA.1 to BA.2, and we cannot predict the direction of its mutation. Therefore, countries need to enhance the global surveillance of coronavirus and its variants with data posted on appropriate reporting platforms (38). For many countries, variants are mainly imported through foreign countries. Thus, countries need to strengthen entry management and the related eradication. Finally, countries should develop regulations related to COVID-19, establish work plans and work coordination mechanisms, clarify responsibilities and the division of labor, and prepare for emergencies.

#### Process

Firstly, countries need to recognize the seriousness of the Omicron variant due to its rapid spread, which requires a faster response (39). In some countries, similar or higher numbers of deaths have been reported when compared to the previous peaks (40). Countries should take an effort to raise awareness and guide people to eliminate fear and cooperate with the implemented measures. Secondly, public health measures such as maintaining social distancing and wearing masks should be adhered to, and restrictive measures such as city closures should be appropriately applied. Public health measures are an effective way of protecting against infection with COVID-19 (41, 42). Thirdly, vaccines are a powerful weapon to prevent COVID-19, and the unvaccinated remain at higher risk of severe disease following infection with Omicron compared to those who have been vaccinated (40). However, vaccine effectiveness decreases over time, and countries should advance sequential immunization, or alternate immunization, to enhance the population’s immune barrier through universal vaccination (20, 43).

#### Outcome

Firstly, countries should strengthen epidemiological investigations, trace close contacts, and take isolation measures. Secondly, countries should promptly conduct multiple rounds of nucleic acid testing. Omicron spreads faster, has less obvious symptom expression and is more insidious, and it is prone to multi-point dissemination and concentrated outbreaks. Only a rapid response and early screening of positive infections to take control measures can try to contain the spread. Moreover, countries should carry out the treatment of patients, strengthen the service capacity of the healthcare system, and promote telemedicine for other patients as well as drug development.

## Conclusion

Based on this study, it seems that China and Israel adopted containment, whereas South Africa and the United States adopted mitigation strategies. A rapid response is a powerful weapon against the Omicron epidemic. To cope with Omicron, we need to recognize its seriousness and firmly take prevention and control measures, especially in terms of immediate collective response in the early stages of the epidemic. Vaccines alone will not get any country out of this crisis, and they should be utilized in coordination non-pharmacological measures (10). According to the SPO model, this is desirable for future work: strengthening emergency management capacity, adhering to public health measures, promoting vaccination, and strengthening patient care and close contact management are the most effective measures in coping with Omicron.

## Data availability statement

The original contributions presented in the study are included in the article/supplementary material, further inquiries can be directed to the corresponding author.

## Author contributions

JJ and GS conceived the manuscript and contributed as the study guarantors. HC, XW, MY, JY, and ML collected the data. JJ drafted the manuscript. LS, HC, and XW revised the manuscript. GS contributed to the critical revision of the manuscript for important intellectual content and approved the final version of the manuscript.

## Funding

This study was supported by the Natural Science Foundation of Guangdong Province in 2022: Construction and application of COVID-19 control model PSR-SOR-Haddon in Guangdong Province (No. 2022A1515011112).

## Conflict of interest

The authors declare that the research was conducted in the absence of any commercial or financial relationships that could be construed as a potential conflict of interest.

## Publisher’s note

All claims expressed in this article are solely those of the authors and do not necessarily represent those of their affiliated organizations, or those of the publisher, the editors and the reviewers. Any product that may be evaluated in this article, or claim that may be made by its manufacturer, is not guaranteed or endorsed by the publisher.

## Footnotes

^1^ http://en.nhc.gov.cn/index.html

^2^ https://www.gov.il/en/departments/ministry_of_health

^3^ https://sacoronavirus.co.za/

^4^ https://www.cdc.gov/coronavirus/2019-ncov/index.html

^5^ https://coronavirus.jhu.edu/?from=groupmessage

^6^ https://covid19.who.int/table
